# Orphan Class A GPCRs Signature Predicts Prognosis and Immune Microenvironment in Gastric Cancer: GPR176 Drives Tumor Progression Through Wnt Signaling and Macrophage Polarization

**DOI:** 10.1155/mi/7977933

**Published:** 2025-07-11

**Authors:** Jiahui Lin, Lingling Ke, Sha Cheng, Wei Lu, Yanan Hu, Xiyi He, Tingting Luo, Yuting Liu, Canxia Xu, Jian Qi

**Affiliations:** ^1^Digestive Diseases Center, Guangdong Provincial Key Laboratory of Digestive Cancer Research, The Seventh Affiliated Hospital, Sun Yat-Sen University, Shenzhen, Guangdong 518107, China; ^2^Department of Gastroenterology, Third Xiangya Hospital of Central South University, Changsha 410013, China; ^3^Department of Pediatric Pulmonology and Immunology, West China Second University Hospital, Sichuan University, Chengdu 610041, China; ^4^Department of Laboratory Medicine, Baiyun District People's Hospital of Guangzhou, Guangzhou 510000, China

**Keywords:** gastric cancer, GPR176, human orphan class A G protein-coupled receptors, macrophage polarization, prognostic model

## Abstract

**Background:** Orphan class A G protein-coupled receptors (GPCRs) are a large and diverse family with broad tissue expression, and their roles in tumors are increasingly recognized. However, their involvement in gastric cancer (GC) remains unclear.

**Methods:** We performed survival and differential expression analyses to characterize orphan class A GPCR expression patterns in stomach adenocarcinoma (STAD). A prognostic risk model was developed using univariate Cox and LASSO regression analysis and validated in the GEO database. Drug sensitivity and immune infiltration were evaluated across different risk groups. The role of GPR176 in GC and its relationship with tumor immunity were further explored using cellular assays.

**Results:** A model incorporating nine orphan class A GPCRs (GPR15, GPR150, GPR176, GPR4, GPR26, GPR78, GPR101, GPR34, and GPR87) was constructed, showing a positive correlation with M2 macrophages and naive B cells. Low-risk patients showed higher sensitivity to AZD6482, BX.795, GDC0941, and pazopanib. GPR176 was found to be upregulated in GC, and functional assays demonstrated that its knockdown suppressed proliferation and migration in the GC cell lines SGC-7901 and HGC-27. GPR176 also modulated the Wnt/β-catenin pathway and M2 macrophage polarization.

**Conclusion:** These findings may provide new insights into the role of orphan class A GPR genes in STAD and identify GPR176 as a new therapeutic target for GC.

## 1. Introduction

Gastric cancer (GC) is the fifth most frequent malignancy and one of the leading causes of cancer deaths worldwide [1]. Its occurrence and progression are controlled by multiple genes and factors [2]. Despite the continuous improvement of clinical treatment and technology, the prognosis for GC is poor due to metastasis, recurrence, and therapy resistance [3]. Therefore, further studies are imperative to understand the pathological mechanism of GC and find new prognostic markers.

G protein-coupled receptors (GPCRs) represent the prominent family of cell surface receptors and regulate diverse physiological functions [4, 5]. Class A orphan receptors are the largest class of GPCRs with a broad tissue distribution, many of which are ubiquitous, and some are specifically expressed in the central nervous system or or immune system. Many orphan GPCRs are conserved across species and are associated with multiple dysfunctions, including brain diseases, cancer, and metabolic disorders [6–9]. However, no studies have yet explored the relevance of class A orphan receptors in GC. Therefore, it is particularly important to explore the function, potential clinical value, and mechanism of class A orphan receptors in GC.

In this study, we used the R language to analyze the mRNA expression matrix of stomach adenocarcinoma (STAD) and normal gastric tissue from the UCSC Xena website to develop a prognostic profile based on class A orphan receptor genes. We also investigated the clinical characteristics, drug sensitivity, and tumor immune infiltration of the analyzed different risk groups. We also found that GPR176 was associated with apoptosis, proliferation, and migration ability of GC cells, and also caused M2 macrophage polarization.

## 2. Materials and Methods

### 2.1. Collection and Analysis of RNA Sequencing Data

The data are available for UCSC Xena (https://xenabrowser.net/datapages/). Data downloaded from GEO (GSE54129 and GSE13911) were used to obtain microarray data of GC. The human orphan class A GPCR gene was obtained from a previous report [10].

### 2.2. Construction of Risk Model

Differentially expressed genes (DEGs) in GC were identified and analyzed using univariate Cox regression to select survival-associated genes. LASSO Cox regression, performed with the “glmnet” R package, was applied to minimize overfitting via k-fold cross-validation, determining the optimal lambda value. Based on this, nine prognostic-related orphan class A GPR genes, GPR15, GPR150, GPR176, GPR4, GPR26, GPR78, GPR101, GPR34, and GPR87, were selected. Subsequently, multivariate Cox proportional hazards regression was used to calculate the regression coefficients of these genes. The risk score for each patient was then computed based on the expression levels and corresponding coefficients of these nine genes using the following formula:

  $$\begin{array}{c} \text{Risk score}=\left(0.125097\times \text{GPR}15\right)+\left(0.049751\times \text{GPR}150\right)+\left(0.170776\times \text{GPR}176\right)+\left(0.201896\times \text{GPR}4\right)+\left(0.188782\times \text{GPR}26\right)+\left(0.371901\times \text{GPR}78\right)+\left(1.0406797\times \text{GPR}101\right)+\left(0.0852064\times \text{GPR}34\right)+\left(0.242414\times \text{GPR}87\right). \end{array}$$

Risk scores were calculated for the TCGA dataset and validated using the GSE84437 dataset. Patients were stratified into high- and low-risk groups based on the median risk score, and survival differences between the two groups were compared using Kaplan–Meier survival analysis.

### 2.3. Construction of Nomogram

A nomogram was developed with the “RMS” R package to predict 1-, 3-, and 5-year survival probabilities in GC patients, with calibration curves plotted for validation.

### 2.4. Drug Sensitivity Analysis

Drug sensitivity scores for small molecules in high- and low-risk groups were calculated using the pRRophetic software package. 3D conformations of relevant drugs were visualized on PubChem (https://pubchem.ncbi.nlm.nih.gov/).

### 2.5. Immune Cell Infiltration Analysis

The R “estimate” package was used to compute StromalScore, ImmuneScore, and ESTIMATEScore. Tumor immune characteristics were studied using the ssGSEA method, and the relationship between GPR176 and immune markers was analyzed using GEPIA (http://gepia.cancer-pku.cn).

### 2.6. Correlations Between GPR176 and m6A Modification in GC

GPR176's association with m6A-related genes (methyltransferases, readers, and demethylases) was analyzed using R, comparing the expression in GC with high and low GPR176 levels.

### 2.7. Construction of lncRNA–miRNA–mRNA ceRNA Network in GC

The miRNAs targeting GPR176 were predicted using microT CDS (www.microrna.gr/microT-CDS), mirDIP (www.ophid.utoronto.ca/mirDIP/), and starBase (www.starbase.sysu.edu.cn). The negatively correlated miRNAs and their target genes were selected from TCGA, and the lncRNAs targeting these miRNAs were predicted using miRNet (https://www.mirnet.ca).

### 2.8. Cell Culture

GES-1 was obtained from Haibo Biotechnology (China), and GC cell lines SGC-7901, HGC-27, and AGS were from Fenghui Biotechnology (China). RAW264.7 macrophages were from Procell (China). Cells were cultured in RPMI-1640 (GES-1, HGC-27) or DMEM (SGC-7901, AGS, RAW264.7) with 10% FBS and 1% antibiotics.

### 2.9. Quantitative Real-Time PCR Analysis

Total cellular RNA was extracted using TRIzol reagent. Reverse transcription was performed using HiScript II Q RT SuperMix for qPCR kit (R223-01, Vazyme, China), and cDNA was synthesized according to the instructions. We use LightCycler 480 Real Time PCR instrument (Roche, Basel, Switzerland) to perform RT-qPCR. The primer sequence is as follows:

  GPR176-forward: 5′-ATGGGGACATAACGGGAGCTG-3′
  GPR176-reverse: 5′-GCGGCAAGTTGACCATAACAC-3′
  GAPDH-forward: 5′-GGTCACCAGGGCTGCTTTA-3′
  GAPDH-reverse: 5′-GGATCTCGCTCCTGGAAGATG-3′.

### 2.10. Western Blot

We lysed cells in RIPA lysis buffer and supplemented with protease inhibitors and phosphatase inhibitors. Cell lysate of protein was separated by SDS-PAGE and transferred to PVDF membranes (Millipore). The primary antibodies GPR176 (1:600 dilution, Bioss), β-catenin (1:1000 dilution, Abmart), cyclin D1 (1:500 dilution, Wanleibio), MMP7 (1:1000 dilution, Cell Signaling Technology), C-Myc (1:500 dilution, Wanleibio), ARG-1 (1:5000 dilution, Proteintech), and GAPDH (1:1000 dilution, Proteintech) were incubated at 4°C overnight. HRP conjugated secondary antibody (Cell Signaling Technology) 1 h at room temperature. Protein bands on developed films were detected, and ImageJ software was analyzed.

### 2.11. Immunohistochemical Staining

From December 2021 to April 2022, we collected the cancer and adjacent tissues of 11 patients with GC who underwent surgical resection in the Department of Gastrointestinal Surgery, Xiangya Third Hospital, Central South University. The tissue samples were embedded in paraffin, sectioned, and used for an immunohistochemical test. Sections were incubated with GPR176 antibody (1:200, Bioss) and HRP-labeled secondary antibody. Staining was visualized using a DAB kit, and the *H*-score was used for analysis. Ethical approval was obtained.

### 2.12. GPR176 Knockdown and Overexpression

Lentiviral vectors targeting GPR176 (shRNA) were purchased from Tsingke Biotech (China). Lentivirus was used to transfect SGC-7901 and HGC-27 cells, and stable cell lines were selected using puromycin (1 μg/mL). Knockdown efficiency was confirmed by Western blotting. For overexpression, the GPR176 plasmid (OV-GPR176) and the empty vector (OV-NC) were transfected into RAW264.7 cells using Lipofectamine 3000 (Thermo Fisher Scientific). Transfection efficiency was assessed by Western blotting. The shRNA sequences used in this part of the study were as follows:

  shGRP176#1: 5′-TCGGAAACTTCATGGTGTTAT-3′
  shGRP176#2: 5′-CCACAGAACACCATCTCTATT-3′
  shGRP176#3: 5′-CGTGTTCAAATCTGTCACCAA-3′
  shGRP176#4: 5′-ATAACATCACCACGGTCATTG-3′

In addition to the design of the blank control, ShNC.

### 2.13. Cell Immunofluorescence

Macrophages were fixed, permeabilized, and incubated with primary antibodies overnight at 4°C. Nuclei were stained with DAPI, and samples were observed under a fluorescence microscope (Carl Zeiss AG, Germany).

### 2.14. Cell Counting Kit-8 (CCK-8) Assays

Cell proliferation was measured using the CCK-8 kit (TargetMol, Cat# C0005). Cells (1 × 10^3^ per well) were incubated with 10 μL of CCK-8 solution, and absorbance at 450 nm was measured after 2 h.

### 2.15. Colony Formation Assay

SGC-7901 and HGC-27 cells (300–500 cells/well) were cultured in 6-well plates for 2 weeks. Colonies were fixed with paraformaldehyde and stained with crystal violet.

### 2.16. Flow Cytometry Analysis

Cells were stained with Annexin V-APC/7-AAD apoptosis kit (Liankebio, Cat# AP105, China). Apoptotic cells were then analyzed using a flow cytometer (BD FACSCanto II, BD Biosciences, USA), and data were processed with FlowJo software (version 10.8.1).

### 2.17. Transwell Assay

Cells were plated in the upper chamber of a Transwell system with serum-free medium, and the lower chamber contained medium with 10% FBS. After 48 h, cells were fixed, stained, and observed under a microscope.

### 2.18. Statistical Analysis

Statistical analyses were conducted using R software (version 4.2.2) and GraphPad Prism (version 9.0). Continuous variables were compared using Student's *t*-test or one-way ANOVA as appropriate. Correlation analyses were performed using Spearman's rank correlation test. For survival analyses, Kaplan–Meier curves with the log-rank test were used, and hazard ratios (HRs) were estimated using univariate and multivariate Cox proportional hazards models. A two-tailed *p* value  < 0.05 was considered statistically significant. Statistical significance is indicated as *⁣*^*∗*^*p* < 0.05, *⁣*^*∗∗*^*p* < 0.01, and *⁣*^*∗∗∗*^*p* < 0.001.

## 3. Results

### 3.1. Identification of Prognosis-Related Orphan Class A GPR Genes in STAD and Their Mutational Landscape

**Figure 1:**
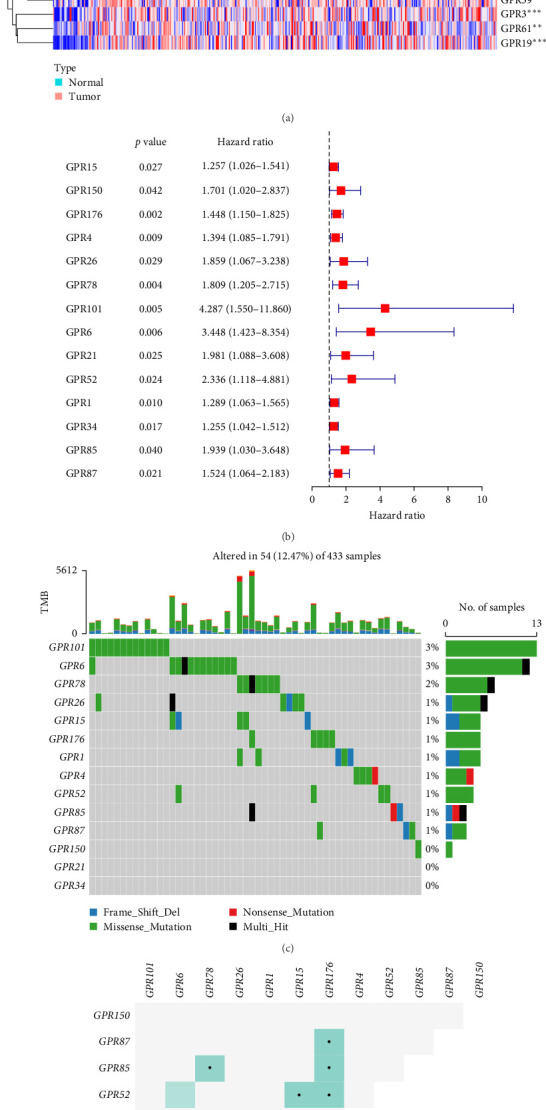
Mutational landscape of the prognosis-related orphan class A GPR gene. (A) Expression profiles of orphan class A GPR genes in tumor and adjacent normal tissues. (B) Univariate Cox regression analysis to map the forest plot of prognosis-related orphan class A GPR genes. (C) Analysis of somatic mutation profiles of orphan class A GPR genes in gastric cancer samples. (D) Co-mutation analysis. *⁣*^*∗*^, *⁣*^*∗∗*^, and *⁣*^*∗∗∗*^ indicate *p* < 0.05, *p* < 0.01, and *p* < 0.001, respectively.

Among the 39 orphan class A GPR genes, 27 exhibited significant differential expression between tumor and adjacent normal tissues (Figure 1A). Univariate Cox regression analysis revealed that 14 of these genes were significantly associated with prognosis in STAD (Figure 1B). Somatic mutation profiles of 433 GC patients, obtained from the TCGA database, were analyzed and visualized using the “maftools” R package. The top 10 mutated orphan class A GPR genes in GC patients were GPR101, GPR6, GPR78, GPR26, GPR15, GPR176, GPR1, GPR4, GPR52, and GPR85, with most mutations being missense (Figure 1C). Co-mutation analysis further identified frequent co-occurrences, including GPR15 with GPR6, GPR15 with GPR78, and GPR1 with GPR78 (*p* < 0.01) (Figure 1D).

### 3.2. Construction and Validation of Features for Orphan Class A GPRs

**Figure 2:**
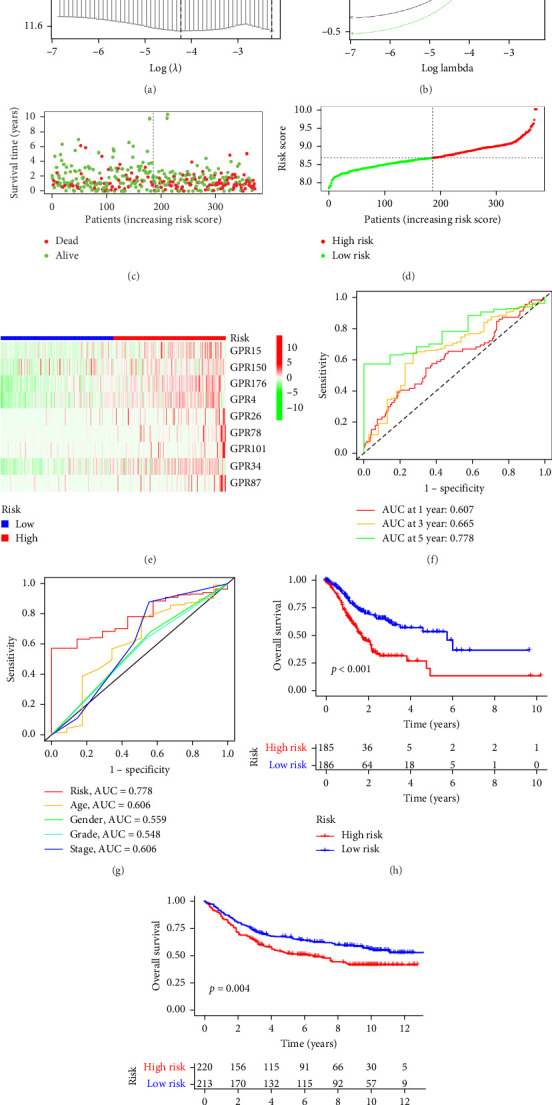
Construction of risk signature. (A) Cross-validation curve. (B) Lasso coefficient path plot. (C, D) Distribution of risk scores and patient survival in two risk subgroups. (E) Heat map of gene expression profiles in different risk sets. (F) Sensitivity and specificity of ROC time curves for predicting OS. (G) ROC curves to assess the sensitivity and specificity of different clinical characteristics and risk scores. (H, I) Overall survival for high and low risk based on TCGA and GSE84437 cohorts, respectively.

Based on univariate Cox regression analysis, we subsequently performed LASSO regression to further refine the prognostic model (Figure 2A,B). The optimal parameter, corresponding to the dashed line on the left side of Figure 2A (lambda.min), was selected, as it yielded the smallest error and identified nine variables for subsequent analysis. These nine orphan class A GPR genes, GPR15, GPR150, GPR176, GPR4, GPR26, GPR78, GPR101, GPR34, and GPR87, were used to construct the prognostic model. Multivariate Cox proportional hazards regression analysis was then applied to calculate the Cox coefficients for these genes. The risk score for each patient was computed based on the expression levels and corresponding coefficients of these nine genes using the following formula:

  $$\begin{array}{c} \text{Risk score}=\left(0.125097\times \text{GPR}15\right)+\left(0.049751\times \text{GPR}150\right)+\left(0.170776\times \text{GPR}176\right)+\left(0.201896\times \text{GPR}4\right)+\left(0.188782\times \text{GPR}26\right)+\left(0.371901\times \text{GPR}78\right)+\left(1.0406797\times \text{GPR}101\right)+\left(0.0852064\times \text{GPR}34\right)+\left(0.242414\times \text{GPR}87\right). \end{array}$$

Patients were stratified into high-risk and low-risk groups based on the median risk score (Figure 2D). Figure 2C illustrates the survival status and duration for patients in the high-risk and low-risk groups. The expression profiles of the nine orphan class A GPR genes are shown in Figure 2E. To evaluate the predictive accuracy of this model, ROC curves were constructed for 1-, 3-, and 5-year overall survival (OS), with the model showing AUC values of 0.607, 0.665, and 0.778, respectively (Figure 2F). Additionally, the area under the ROC curve (AUC) values of the risk score were compared with those for age, gender, stage, and grade at 5 years of OS. The results demonstrated that the risk score had superior AUC values and a better predictive capability than other clinical factors (Figure 2G). Furthermore, patients in the high-risk group exhibited significantly higher mortality rates and shorter survival times compared to those in the low-risk group (*p* < 0.001) (Figure 2H). The model was validated using the GSE84437 cohort, and Kaplan–Meier analysis revealed a significant survival difference between the low-risk and high-risk groups in this cohort (*p* = 0.004) (Figure 2I).

### 3.3. Development and Validation of a Nomogram Based on Prognostic Characteristics of Orphan Class A GPRs

**Figure 3:**
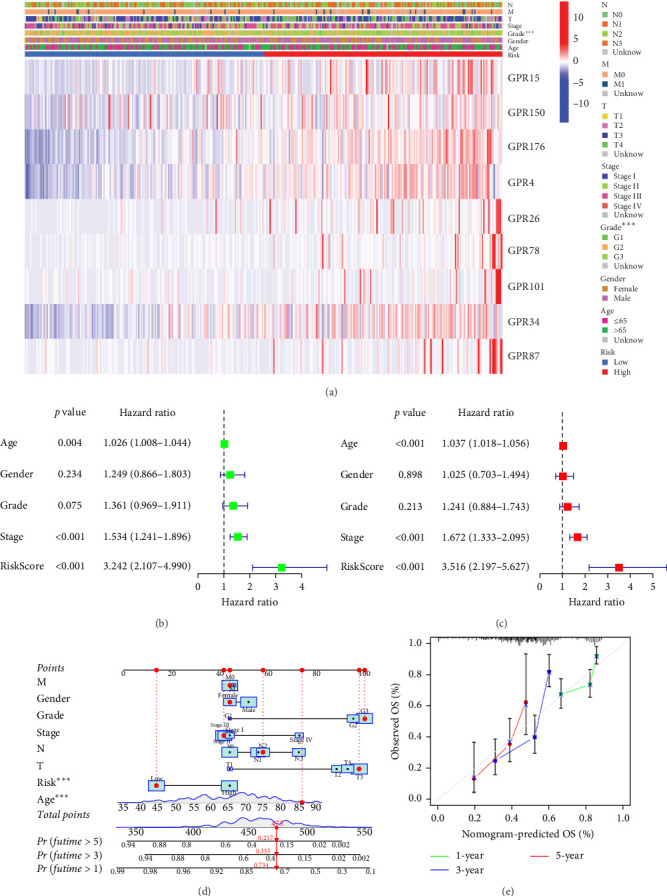
Assessment of prognostic features and nomogram construction of orphan class A GPRs in STAD. (A) Association between risk scores and different clinicopathological characteristics. (B, C) Univariate and multivariate Cox regression analysis based on risk scores and clinical characteristics of STAD patients. (D) Nomogram combining risk scores and clinicopathological characteristics. (E) Calibration curves for column line plots.

To comprehensively assess the prognostic value of the model, we next examined the relationship between clinicopathological characteristics and the risk score model. A chi-square test was conducted to determine whether the prognostic characteristics of orphan class A GPRs were predictive of clinical and pathological variables in STAD. The results revealed a significant difference in the distribution of tumor grade between the low-risk and high-risk groups (*p* < 0.001) (Figure 3A). Furthermore, univariate Cox regression analysis indicated that the prognostic characteristics of orphan class A GPRs were significantly associated with poor OS (HR: 3.242, 95% CI: 2.107–4.990, *p* < 0.001) (Figure 3B). Multivariate Cox regression analysis confirmed that age, grade, and risk score (RiskScore) were independent prognostic factors for OS (Figure 3C). Based on these findings, we developed a nomogram incorporating RiskScore and clinicopathological characteristics to predict 1-, 3-, and 5-year survival in STAD (Figure 3D). Calibration plots for 1-, 3-, and 5-year survival predictions demonstrated good agreement between the nomogram's predictions and actual outcomes (Figure 3E).In conclusion, the RiskScore proved to be a reliable and effective tool for prognostic prediction in STAD, highlighting its potential clinical utility.

### 3.4. Assessment of Immune Cell Infiltration and Immunotherapy Response Based on Risk Score

**Figure 4:**
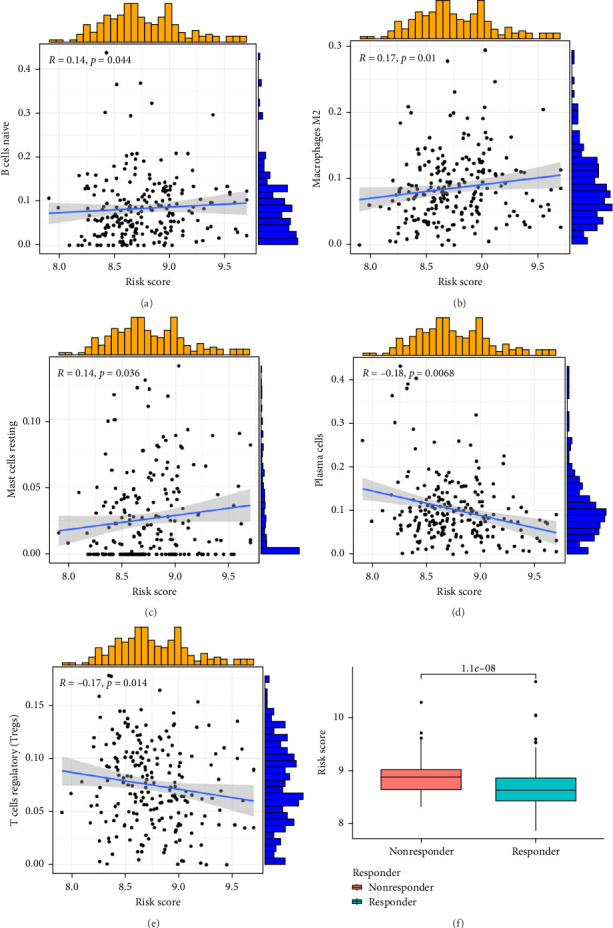
Association of risk scores with immune cell infiltration and immunotherapy response. (A–E) Correlation between the risk score and the infiltration levels of naive B cells, M2 macrophages, resting mast cells, plasma cells, and regulatory T cells (Tregs), respectively, as estimated by the CIBERSORT algorithm. (F) Differences in predicted immunotherapy response between high- and low-risk groups.

To investigate the relationship between orphan class A GPR-based risk scores and the tumor immune microenvironment, we first applied the CIBERSORT algorithm. We observed that higher risk scores were significantly positively correlated with naive B cells, M2 macrophages, and resting mast cells (*p* < 0.05; Figure 4), while they were significantly negatively correlated with plasma cells and regulatory T cells (Tregs) (*p* < 0.05; Figure 4D,E). In addition, we examined the predicted immunotherapy response in the two risk groups. Patients with higher risk scores were less likely to respond to immune checkpoint blockade therapies (Figure 4F), suggesting that high-risk individuals may have a more immunosuppressive microenvironment.

### 3.5. Drug Sensitivity Prediction and Potential Therapeutic Compounds in High- and Low-Risk Groups

**Figure 5:**
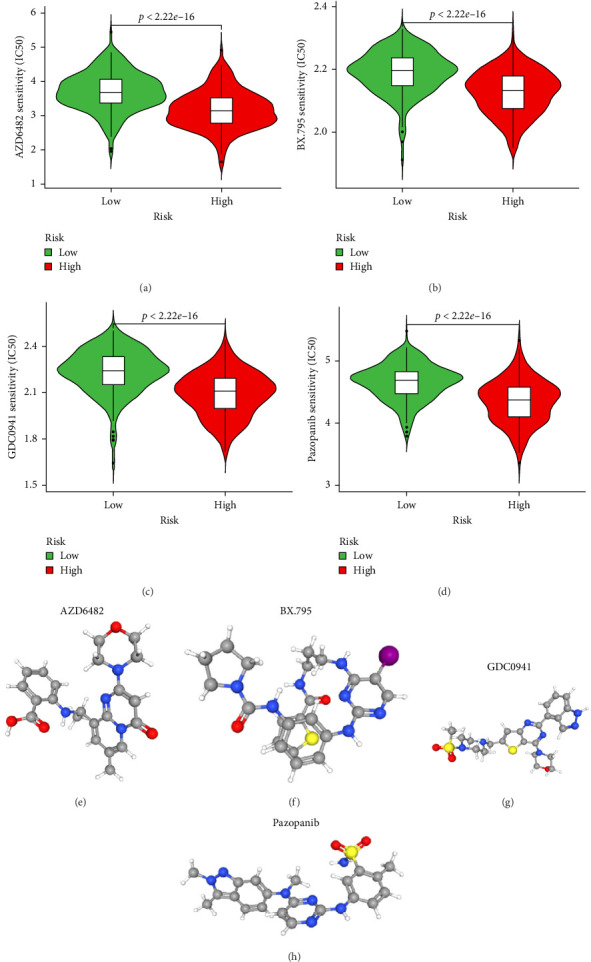
Prediction of drug sensitivity and structural visualization of candidate compounds. (A–D) Estimated IC50 values of AZD6482, BX.795, GDC0941, and pazopanib between high- and low-risk STAD patient groups. (E–H) 3D molecular structures of AZD6482, BX.795, GDC0941, and pazopanib obtained from PubChem.

To further explore treatment strategies, we predicted the chemotherapy response based on risk stratification using the pRRophetic algorithm and IC50 data from the Genomics of Drug Sensitivity in Cancer (GDSC) database. A total of 87 small-molecule compounds showed significantly different sensitivities between high- and low-risk groups. Among them, four compounds, AZD6482 (PI3K inhibitor), BX.795 (TBK1 inhibitor), GDC0941 (dual PI3K/mTOR inhibitor), and pazopanib (tyrosine kinase inhibitor), exhibited the most significant differences (*p* < 2.22e–16; Figure 5A–D). Patients in the low-risk group were more sensitive to these agents, indicating a potentially better therapeutic window. The 3D conformations of these compounds were retrieved from the PubChem database and are shown in Figure 5. These results suggest candidate drugs for personalized chemotherapy in GC based on molecular risk profiling.

### 3.6. Prognostic Analysis of GPR176 in GC

In the TCGA-STAD cohort, high expression of GPR176 was significantly associated with poor OS (HR = 1.69, 95% CI: 1.21–2.37, *p* = 0.002). Further validation through TIMER and GEPIA analyses confirmed that GPR176 overexpression correlated with adverse prognosis in GC, with HR values of 1.18 (*p* = 0.0308) and 1.6 (*p* = 0.0048), respectively. Kaplan–Meier database analysis demonstrated that high GPR176 expression was strongly associated with worse OS, progression-free survival (PFS), and post-progression survival (PPS), with HRs of 1.55, 1.58, and 1.87, respectively. Subgroup analyses revealed that high GPR176 expression correlated with poor prognosis in intestinal-type GC, advanced T and N stages, and female patients (Figure S1).

Cox regression analysis indicated that univariate analysis identified high GPR176 expression (*p* = 0.002), N stage (*p* = 0.002), M stage (*p* = 0.004), pathological stage (*p* < 0.001), age (*p* = 0.005), and T stage (*p* = 0.011) as significantly associated with poor OS. In multivariate Cox regression, high GPR176 expression (*p* = 0.002), M stage (*p* = 0.016), and age (*p* = 0.002) were identified as independent prognostic factors for poor prognosis in GC patients. These findings further support GPR176 as an independent prognostic biomarker for GC (Table S1).

### 3.7. GSEA Analysis Between Low and High GPR176 Expression

In order to analyze the molecular function of GPR176 in GC, we performed GSEA analysis between low and high GPR176 expression to predict GPR176-related signal pathways. Terms that are significantly enriched in cell adhesion and tumourigenesis included “Wnt signaling pathway,” “ECM–receptor interaction,” “focal adhesion,” and “extracellular matrix–receptor interaction”. The related terms involved in immune and inflammatory reactions included “T toll receptor pathway,” “TGF-β signaling pathway,” “Toll-like receptor signaling pathway,” “JAK-STAT signaling pathway,” and “cytokine signaling pathway” (Figure S2).

### 3.8. The Correlations Between GPR176 and Immune Cell Infiltration

We analyzed the StromalScore, ImmuneScore, and ESTIMATEScore of tumor samples with high and low GPR176 expression. The results showed that the high-expression group had significantly higher scores than the low-expression group (Figure S3 A–C). Significant differences in immune cell expression were observed between the two groups, including macrophages, TEM cells, and NK cells (*p* < 0.05) (Figure S3D). ssGSEA and Spearman correlation analyses indicated that GPR176 was positively correlated with multiple immune cell types, particularly macrophages (*r* = 0.494, *p* < 0.001), and negatively correlated with Th17 cells (Figure S3F). TIMER analysis further revealed a significant association between GPR176 and the infiltration of CD8 ^+^ T cells, CD4^ +^ T cells, and macrophages (Figure S4A). Genes with somatic cell copy number change are markers of tumourigenesis and progression [11], and may affect the response of immunotherapy [12]. Therefore, the SCNA module was used to analyze the correlation between the change of gene copy number of GPR176 and the infiltration level of immune cells. The SCNA module analysis demonstrated that copy number variations in GPR176 affected the infiltration of immune cells, especially CD8 ^+^ T cells, B cells, and macrophages (Figure S4B). Moreover, the mutation frequency of the GPR176 gene was notably higher in GC (Figure S4C), and high macrophage infiltration was associated with poor prognosis in GC (*p* = 0.004) (Figure S4D). GPR176 was strongly correlated with tumor-associated macrophages (TAMs) and M2 macrophage marker genes, suggesting its crucial role in the immune microenvironment of GC.

We conducted an in-depth investigation into the expression of GPR176 and its association with the tumor immune system in human cancers. We found a significant correlation between GPR176 expression and several immune-related molecules in STAD. Specifically, the expression of GPR176 was positively correlated with the following molecules: TGFB1 (transforming growth factor beta 1), an immunosuppressive factor (*r* = 0.498, *p* < 2.2e–16); ENTPD1 (ectonucleoside triphosphate diphosphohydrolase 1), an immune-stimulating factor (*r* = 0.508, *p* < 2.2e–16); CXCL12 (CXC motif chemokine ligand 12), a chemokine (*r* = 0.478, *p* < 2.2e–16); and CCR4 (C–C chemokine receptor type 4), a chemokine receptor (*r* = 0.401, *p* < 2.2e–16) (Figure S5). Based on these results, we hypothesize that the overexpression of GPR176 might exert a suppressive effect on cancer progression by modulating the activity of immune-stimulating factors, such as ENTPD1. Moreover, GPR176 may influence the immune status of the tumor microenvironment in STAD by regulating the immunosuppressive function of TGFB1 and the chemotactic activity of CXCL12, further affecting tumor progression. These findings suggest that GPR176 may play a significant role in the immune regulation of STAD, providing a theoretical foundation for future research exploring GPR176 as a potential therapeutic target.

### 3.9. Correlation Between GPR176 and m6A Methylation

We found that GPR176 was positively correlated with m6A relative genes, including methyltransferases (WTAP, METTL3, and METTL14, etc.), demethylases (ALKBH5 and FTO), and methylated reading proteins (IGF2BP3, YTHDC1-2, and YTHDF1-3, etc.) (Figure S6A). Figure S6B shows the m6A methylation-related genes with the top 4 correlations, among which FTO has the most significant relationship with GPR176 (*r* = 0.473, *p* < 0.001).

### 3.10. Construction of ceRNA Regulatory Network of GPR176 in GC

In this study, we constructed a ceRNA regulatory network for GPR176 in GC. Using the microT CDS, mirDIP, and starBase tools, we predicted 114, 692, and 96 miRNAs targeting GPR176, respectively (Figure S7A). Based on these 25 predicted miRNAs, we established the ceRNA network (Figure S7B). Further analysis revealed that hsa-miR-144-3p was negatively correlated with GPR176 and significantly downregulated in GC (Figure S7E–H).

We then employed starBase and miRNet to predict potential lncRNA binding partners of hsa-miR-144-3p, and the results were visualized using a Venn diagram (Figure S7C). A ceRNA network was constructed based on these lncRNAs (Figure S7D). The analysis showed that lncRNAs, such as ZNF460-AS1, THUMPD3-AS1, and SNHG14, were negatively correlated with hsa-miR-144-3p in GC (Figure S7G). Among these, 12 lncRNAs were positively correlated with GPR176 expression. Further investigation revealed that eight of these lncRNAs were highly expressed in GC (Figure S8). Prognostic analysis of these eight lncRNAs indicated that only LINC00662 was significantly associated with poor prognosis in GC (Figure S7J). The binding sites of hsa-miR-144-3p on GPR176 and LINC00662 are shown in Figure S7K. In conclusion, we constructed the LINC00662-hsa-miR-144-3p-GPR176 ceRNA network in GC, providing insights into its potential role in the disease.

### 3.11. GPR176 is Highly Expressed in GC

**Figure 6:**
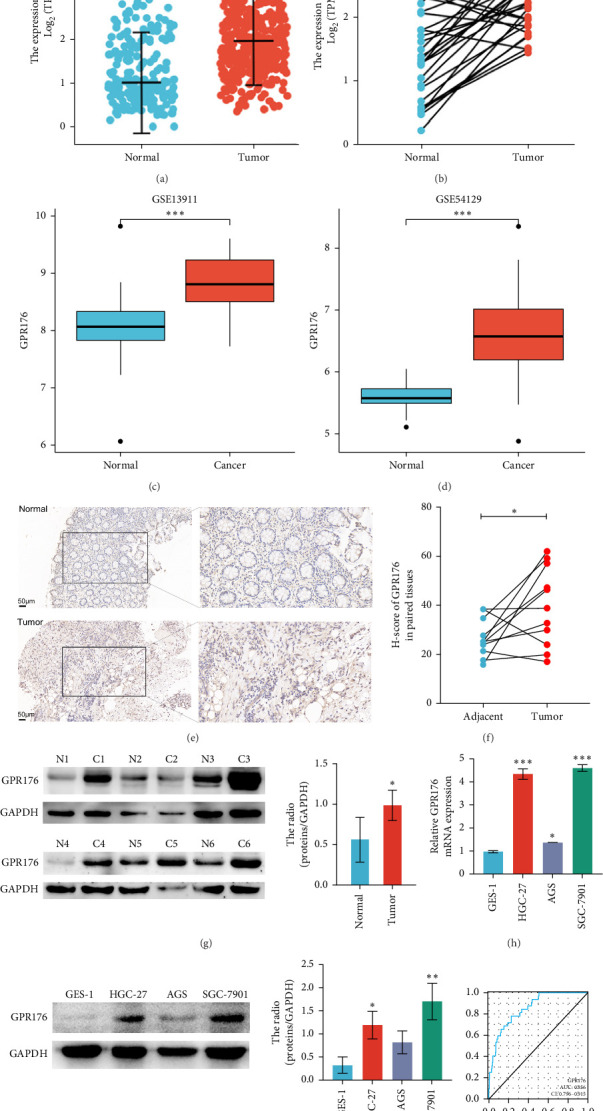
Expression levels of GPR176 in GC. (A) The expression of GPR176 in TCGA_GTEx-STAD. (B) Expression of GPR176 in matched tumor samples. (C, D) The expression of GPR176 in GSE13911 and GSE54129. (E) Typical immunohistochemical picture of GPR176 protein. Score bars, 200, 50 μm. (F) Quantitative analysis of immunohistochemistry. (G) Western blot showed that the level of GPR176 protein was significantly higher in the 6 gastric cancer tissues than in the adjacent gastric mucosal tissues, “C” represents GC tissues, and “N” represents adjacent tissues. (H, I) Western blot detection and qRT-PCR detection of mRNA expression levels of GPR176 in Ges-1 and different GC cell lines. (J) ROC curve analysis of GPR176 diagnosis. *⁣*^*∗*^, *⁣*^*∗∗*^, and *⁣*^*∗∗∗*^ indicate *p* < 0.05, *p* < 0.01, and *p* < 0.001, respectively.

In the TCGA_GTEx-STAD dataset, GPR176 mRNA expression was significantly higher in 414 GC samples compared to 210 normal samples (Figure 6A). In matched pair analysis, GPR176 expression was notably elevated in 32 tumor samples compared to adjacent normal gastric tissues (Figure 6B). Further validation using GEO databases confirmed the upregulation of GPR176 in GC, with significant increases observed in the GSE54129 and GSE13911 cohorts (Figure 6C,D). To validate these findings, we collected 11 pairs of GC clinical specimens and assessed GPR176 expression in both cancerous and adjacent normal tissues using immunohistochemical staining. The results revealed a significant increase in GPR176 expression in GC tissues (Figure 6E,F). Western blot analysis of 6 paired GC tissue samples further confirmed the marked upregulation of GPR176 in GC (Figure 6G). Additionally, we evaluated GPR176 expression in normal gastric epithelial cells and various GC cell lines (HGC-27, AGS, SGC-7901) through QPCR and Western blot. We found that GPR176 was significantly upregulated at both the RNA and protein levels in all GC cell lines (Figure 6H,I), with higher expression observed in HGC-27 and SGC-7901 cells compared to AGS cells. Finally, we performed ROC curve analysis to assess the diagnostic potential of GPR176 expression in distinguishing GC from normal tissues. The area under the ROC curve (AUC) was 0.856, indicating strong predictive accuracy (Figure 6J).

### 3.12. GPR176 Regulates the Malignant Phenotype of GC Cells and Promotes M2 Macrophage Polarization

**Figure 7:**
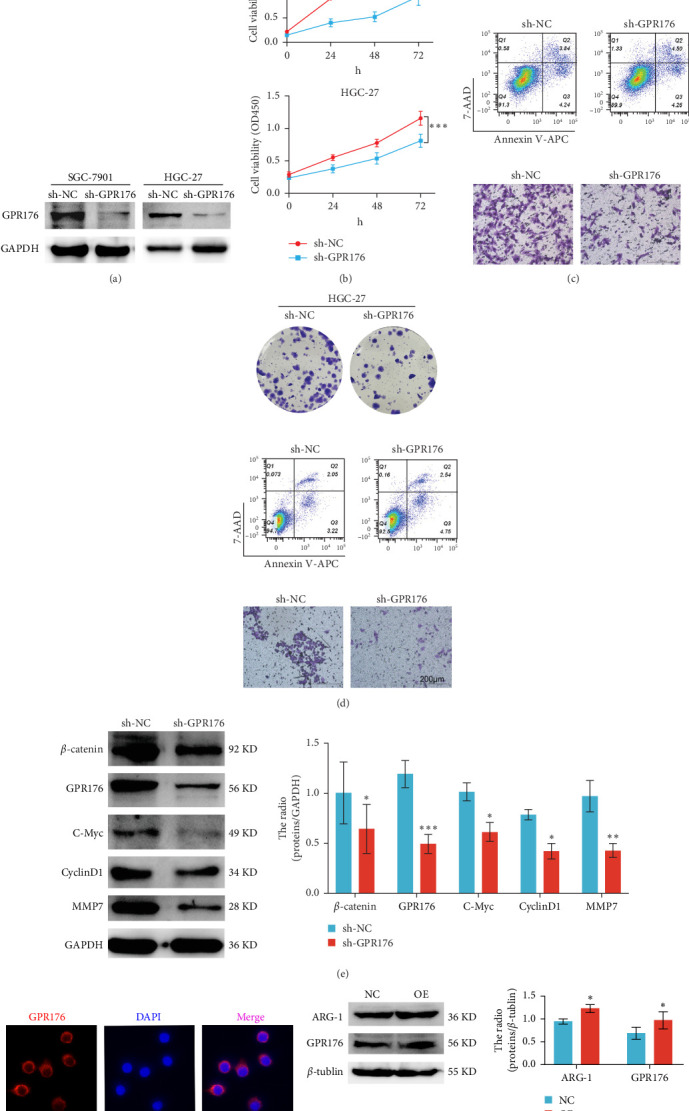
GPR176 regulates the malignant phenotype of gastric cancer cells and promotes M2 macrophage polarization. (A) The transfection efficiency of sh-GPR176 in SGC-7901 and HGC-27 cell lines was investigated by WB. (B) CCK-8 assay was used to detect the effect of GPR176 knockdown on the proliferation of SGC-7901 and HGC-27 cell lines. (C, D) The results of colony formation, apoptosis, and transwell assays in SGC-7901 and HGC-27 cell lines following GPR176 knockdown. Score bars, 200 μm. (E) Expression of β-catenin, GPR176, C-Myc, cyclin D1, and MMP7 was detected by WB. (F) Immunofluorescence staining results showed that GPR176 was mainly distributed on the cell membrane in macrophages. Scale bar, 5 μm. (G) Expression of ARG-1, GPR176, and β-tublin.*⁣*^*∗*^, *⁣*^*∗∗*^, and *⁣*^*∗∗∗*^ indicate *p* < 0.05, *p* < 0.01, and *p* < 0.001, respectively.

Knockdown of GPR176 significantly inhibits the malignant phenotype of GC cells. We employed specific shRNAs to knock down GPR176 expression in the SGC-7901 and HGC-27 cell lines, and Western blot analysis confirmed a marked reduction in GPR176 expression (Figure 7A). CCK-8 assays showed that GPR176 knockdown significantly suppressed cell proliferation (Figure 7B), and the colony formation assay further validated this result, with colonies formed by sh-GPR176-transfected cells being significantly smaller than those in the sh-NC group. Flow cytometry analysis revealed a significant increase in apoptosis in both SGC-7901 and HGC-27 cells following GPR176 knockdown, while Transwell assays demonstrated a notable reduction in cell migration (Figure 7C,D).

Moreover, GPR176 regulates the Wnt/β-catenin signaling pathway. In SGC-7901 cells with GPR176 knockdown, expression of β-catenin, MMP7, C-Myc, and Cyclin D1 was significantly decreased (Figure 7E), suggesting that GPR176 exerts its effects on GC cells through modulation of the Wnt signaling pathway and cell cycle-related proteins. Immune cell infiltration analysis revealed a significant association between GPR176 and M2 macrophage infiltration. Immunofluorescence staining showed that GPR176 was primarily localized to the cell membrane of macrophages (Figure 7F), and overexpression of GPR176 in macrophages resulted in a marked increase in the expression of the M2 macrophage marker ARG-1 (Figure 7G), indicating that GPR176 promotes macrophage polarization toward the M2 phenotype.

## 4. Discussion

The role of human orphan class A GPCRs in pathogenesis of various cancers has attracted more and more attention. We analyze functional roles of orphan class A GPRs in GC and identify GPR176 as novel therapy target for GC.

We constructed a prognostic risk model by univariate Cox and Lasso Cox regression analysis using nine genes (GPR15, GPR150, GPR176, GPR4, GPR26, GPR78, GPR101, GPR34, and GPR87). GPR15 controls the specific homing of T cells, especially FOXP3 Tregs, to the lamina propria of the large intestine (LILP) [13]. GPR15 promotes colorectal cancer by promoting the recruitment of Tregs [14]. Aberrant methylation of GPR150 may be a candidate tumor marker for ovarian cancer [15]. GPR176 promotes colorectal carcinoma progression by interacting with the G protein GNAS [9]. GPR4 is a member of the proton-sensing GPRs [16]. In the tumor microenvironment, mesenchymal acidic pH can activate GPR4 to regulate the behavior of tumor cells [17, 18]. Ubiquitin-dependent degradation regulates the constitutive activity of GPR26 has been studied in hepatocellular carcinoma [19]. GPR78 can promote lung cancer cell migration and metastasis by activating the Gαq-Rho GTPase pathway [20]. GPR101 has been reported to play an important role in pituitary tumors in several studies [21, 22]. GPR34 translocations and mutations are associated with salivary gland MALT lymphoma specificity [23]. GPR87 is specifically expressed in tumor cells and is rarely expressed in normal cells [24], and it is essential for p53-dependent cell survival in response to DNA damage [25]. All of the above studies demonstrate the importance of these genes in tumor development. The results of survival analysis showed that the prognosis of the high-risk group was worse than that of the low-risk group, and the risk score was an independent predictor of OS.

TME is composed of many factors [26, 27], which can affect the tumor response to immunotherapy [28]. We explored the relationship between risk scores and naive B cells, M2 macrophages, resting mast cells, plasma cells, and Tregs. It is suggested that the high infiltration level of M2 macrophages and TAM may be a bad prognostic factor for GC patients [29, 30]. The results of our analysis found that the risk scores of orphan class A GPRs were positively correlated with M2 macrophages, suggesting an important role for M2 macrophages. In addition, we screened four potential small-molecule compounds for GC, including AZD6482 (PI3K inhibitor), BX.795 (TBK1 inhibitor), GDC0941 (dual PI3K and mTOR inhibitor), and pazopanib (tyrosine kinase inhibitor). These potential agents may provide new insights into the treatment of GC patients with different risk scores.

GPR176 is one of the most important model genes in our study, and it has a significant prognostic value. Therefore, we focused more on the role of GPR176 in GC. First, we observed higher expression of GPR176 in GC tissues. Then we investigated the functional role of GPR176 in GC cell lines (SGC-7901 and HGC-27). The results showed that GPR176 knockdown inhibited the proliferation and migration of GC cells and promoted their apoptosis. GSEA analysis indicated that GPR176 was significantly related to Wnt signaling pathways. This pathway has been found to be correlated with the progression and metastasis of GC [31]. In the present study, we found that downregulation of GPR176 decreased the expression of β-catenin and downstream target genes, such as cyclin D1, C-Myc, and MMP-7. β-catenin is a key target molecule in the Wnt pathway. β-catenin's active form is dephosphorylated β-catenin, which translocates to the nucleus and stimulates the expression of Wnt target molecules, such as cyclin D1 and MMP-7. The active form of β-catenin is dephosphorylated β-catenin, which translocates to the nucleus and stimulates the expression of Wnt target molecules, such as D1 and MMP-7. MMP-7 belongs to the matrix metalloproteinase family and plays a critical role in the invasion and growth of gastric adenocarcinoma by degrading various protein components of the extracellular matrix [32]. Thus, GPR176 could act as an oncogene in GC by activating the Wnt signaling pathway.

Last but not least, we revealed a potential regulatory role of GPR176 on TAM polarization. In our research, we found that the increase in macrophage infiltration level is related to the poor prognosis of GC patients. We hypothesized that GPR176 might affect the progression of GC by promoting macrophage polarization and inhibiting antitumor immunity. This may explain, to some extent, why the prognosis of GC with high expression of GPR176 is poor.

This research is the first to explore the relationship between human orphan class A GPRs and GC. We systematically analyzed and used different databases for cross-validation, but there are some limitations. First, further validation studies are needed through a larger patient cohort and long-term follow-up to explicitly validate. Second, lack of in vivo xenograft tumor experiments to validate the relationship between GPR176 and cellular biological behavior.

## 5. Conclusions

We constructed a prognostic risk model based on the human orphan class A GPCR gene and identified GPR176 as a new therapeutic target for GC.

## Data Availability

All datasets analyzed in this study are publicly available through the UCSC Xena platform (https://xenabrowser.net/datapages/) and the GEO database (accession numbers: GSE54129, GSE13911, and GSE84437). Additional data supporting the findings are available from the corresponding authors upon reasonable request.

## Supporting Information

Supporting Information Table S1: Cox regression analysis of STAD in TCGA. Figure S1: Prognostic relationship between GPR176 and GC. Figure S2: Enrichment analysis of GPR176 gene set. Figure S3: Analysis of the relationship between GPR176 expression and immune cell infiltration based on TCGA database. Figure S4: The relationship between GPR176 and immune cell infiltration was analyzed based on TIMER database. Figure S5: GPR176 is related to immunomodulators and chemokines in GC. Figure S6: Correlation analysis between GPR176 and m6A methylation. Figure S7: Prediction and construction of GPR176 ceRNA network in GC. Figure S8: Construct LINC00662-has-mir-144-3 p-GPR176 ceRNA network in GC.
