# Supplementary material for: Orphan Class A GPCRs Signature Predicts Prognosis and Immune Microenvironment in Gastric Cancer: GPR176 Drives Tumor Progression Through Wnt Signaling and Macrophage Polarization
Source: Mediators Inflamm. 2025 Jul 11;2025:7977933. doi: 10.1155/mi/7977933 (PMC12274089; doi:10.1155/mi/7977933)
Supplement: Supporting Information — Table S1: Cox regression analysis of STAD in TCGA. Figure S1: Prognostic relationship between GPR176 and GC. Figure S2: Enrichment analysis of GPR176 gene set. Figure S3: Analysis of the relationship between GPR176 expression and immune cell infiltration based on TCGA database. Figure S4: The relationship between GPR176 and immune cell infiltration was analyzed based on TIMER database. Figure S5: GPR176 is related to immunomodulators and chemokines in GC. Figure S6: Correlation analysis between GPR176 and m6A methylation. Figure S7: Prediction and construction of GPR176 ceRNA network in GC. Figure S8: Construct LINC00662-has-mir-144-3 p-GPR176 ceRNA network in GC. [file 7977933.f1.docx]

Supplementary Table 1 Cox regression analysis of STAD in TCGA

| **Characteristics** | **Total(N)** | **Univariate analysis** | |  | **Multivariate analysis** | |
| --- | --- | --- | --- | --- | --- | --- |
|  |  | **Hazard ratio (95% CI)** | **P value** |  | **Hazard ratio (95% CI)** | **P value** |
| **N stage** | **352** |  |  |  |  |  |
| **N0** | **107** | **Reference** |  |  |  |  |
| **N1&N2&N3** | **245** | **1.925 (1.264-2.931)** | **0.002** |  | **1.557 (0.870-2.787)** | **0.136** |
| **M stage** | **352** |  |  |  |  |  |
| **M0** | **327** | **Reference** |  |  |  |  |
| **M1** | **25** | **2.254 (1.295-3.924)** | **0.004** |  | **2.108 (1.151-3.860)** | **0.016** |
| **Pathologic stage** | **347** |  |  |  |  |  |
| **Stage I&Stage II** | **160** | **Reference** |  |  |  |  |
| **Stage III&Stage IV** | **187** | **1.947 (1.358-2.793)** | **<0.001** |  | **1.240 (0.719-2.138)** | **0.440** |
| **Age** | **367** |  |  |  |  |  |
| **<=65** | **163** | **Reference** |  |  |  |  |
| **>65** | **204** | **1.620 (1.154-2.276)** | **0.005** |  | **1.796 (1.245-2.592)** | **0.002** |
| **Gender** | **370** |  |  |  |  |  |
| **Female** | **133** | **Reference** |  |  |  |  |
| **Male** | **237** | **1.267 (0.891-1.804)** | **0.188** |  |  |  |
| **T stage** | **362** |  |  |  |  |  |
| **T1&T2** | **96** | **Reference** |  |  |  |  |
| **T3&T4** | **266** | **1.719 (1.131-2.612)** | **0.011** |  | **1.172 (0.703-1.955)** | **0.543** |
| **GPR176** | **370** |  |  |  |  |  |
| **Low** | **185** | **Reference** |  |  |  |  |
| **High** | **185** | **1.690 (1.206-2.367)** | **0.002** |  | **1.801 (1.252-2.591)** | **0.002** |

Supplementary Figure1


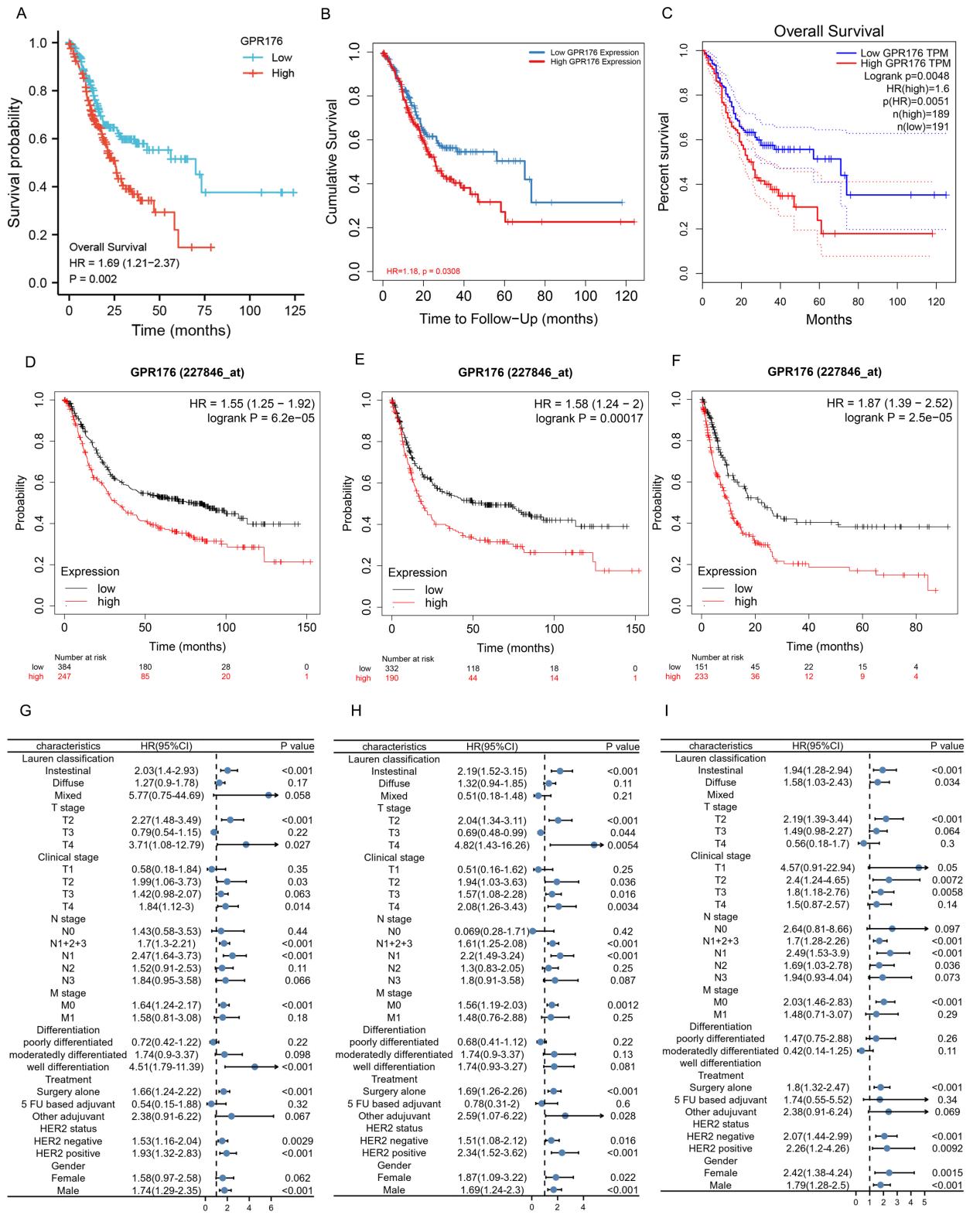


**Supplementary Figure1 Prognostic relationship between GPR176 and GC (A-C)** Analysis of the relationship between GPR176 expression and OS based on the TCGA database, TIMER database and GEPIA database, respectively. **(D-F)** The relationship between GPR176 expression and OS, FPS and PPS based on Kaplan-Meier database. **(G-I)** Based on Kaplan-Meier database, the relationship between GPR176 expression and OS, FPS and PPS among subgroups were shown by forest diagram.

Supplementary Figure2


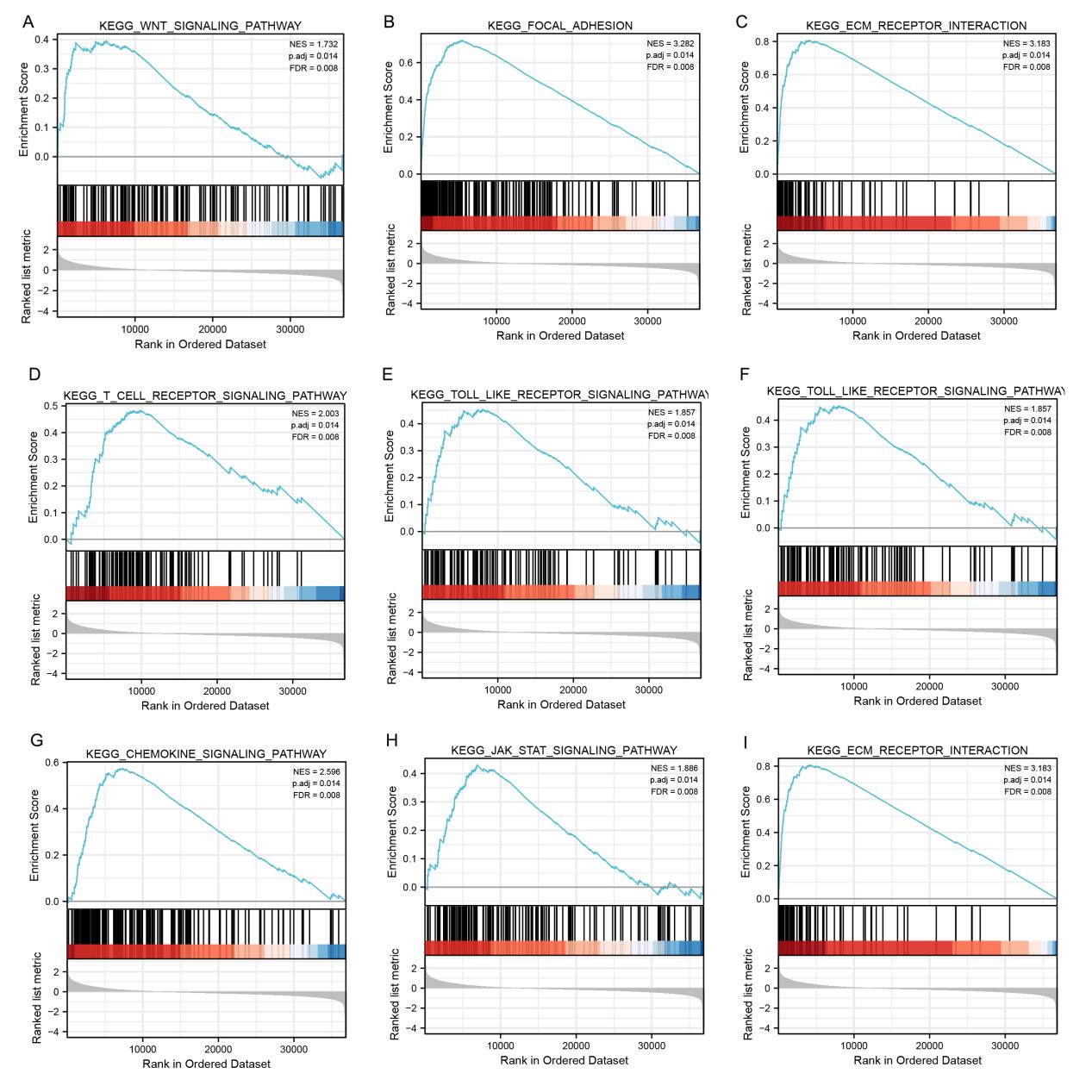


**Supplementary Figure2. Enrichment analysis of GPR176 gene set** (A) WNT signalling pathway (B) focal adhesion (C) Extracellular matrix-receptor interaction (D) T cell receptor pathway (E) Toll-like receptor signalling pathway (F) TGF-β signalling pathway (G) Cytokine signalling pathway (H) JAK-STAT signalling pathway. (I) ECM-receptor-interaction. NES, normalized enrichment score; FDR, false discovery rate; *p*. adj, adjusted *p* value.

Supplementary Figure3


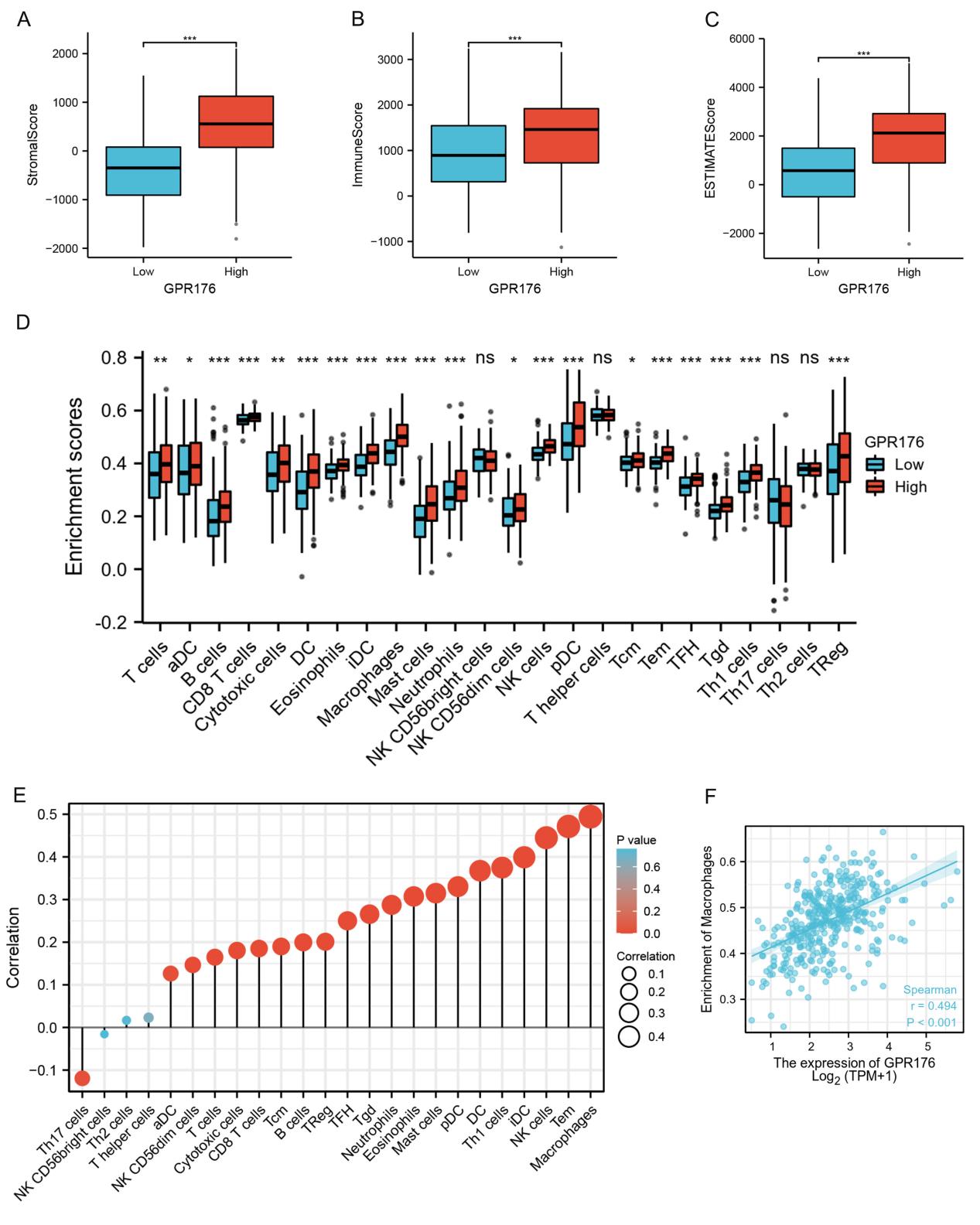


**Supplementary Figure3. Analysis of the relationship between GPR176 expression and immune cell infiltration based on TCGA database** (A-C) StromalScore, ImmuneScore and ESTIMATEScore of each tumour sample between the high and low expression GPR176 groups (D) Infiltration of immune cells between high and low expression GPR176 groups. (E) Lollipop chart shows the correlation analysis between GPR176 and immune cell infiltration degree (F) The correlation between GPR176 and the infiltration level of macrophages

Supplementary Figure4


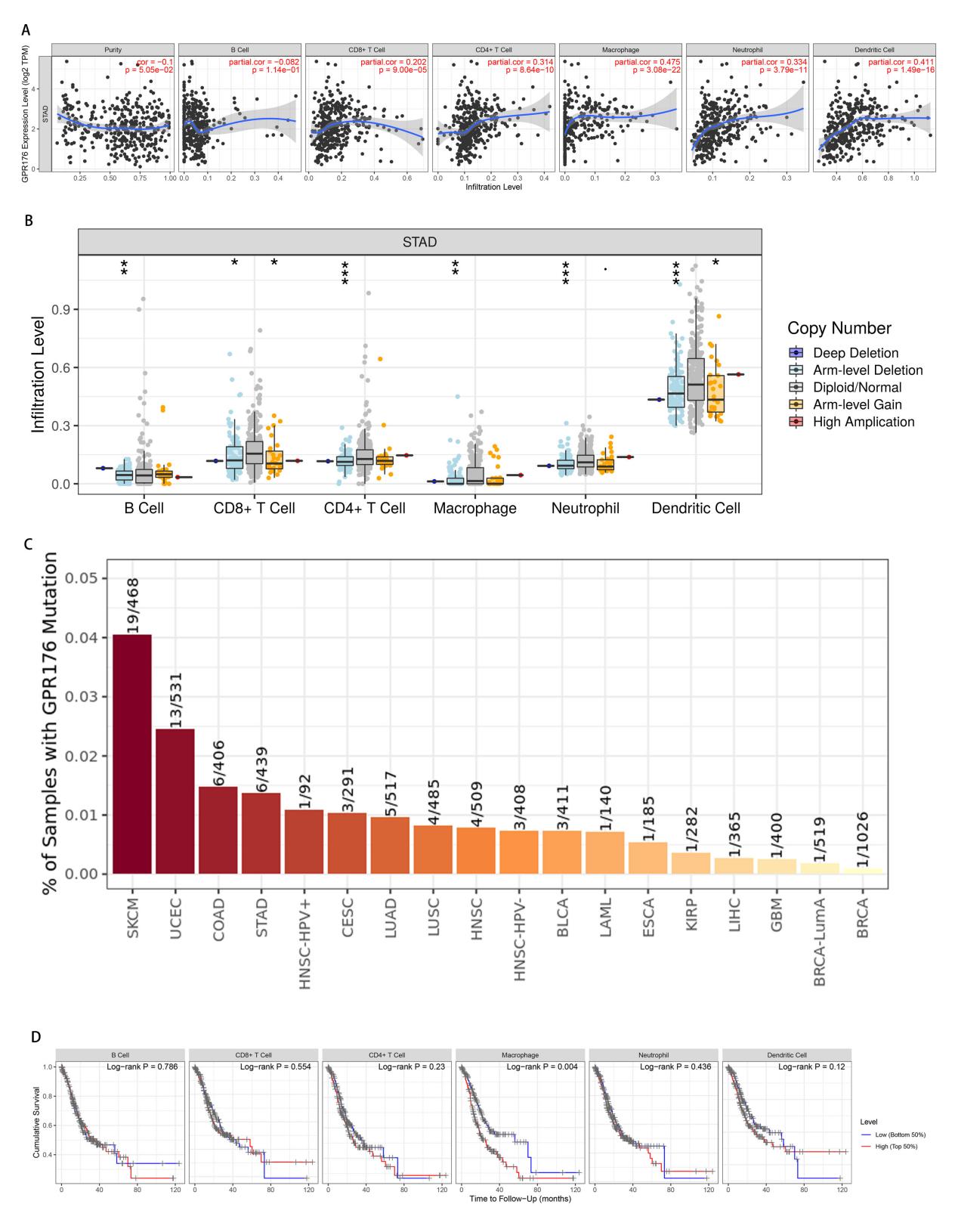


**Supplementary Figure S4. The relationship between GPR176 and immune cell infiltration was analyzed based on TIMER database** (A) Correlation between GPR176 and immune infiltration level (B) Relationship between copy number change of GPR176 gene and infiltration level of immune cells (C) Mutation module analyzes the gene mutation frequency of GPR176 in TCGA cancer types (D) Correlation between immune infiltration level and prognosis of patients.

Supplementary Figure5


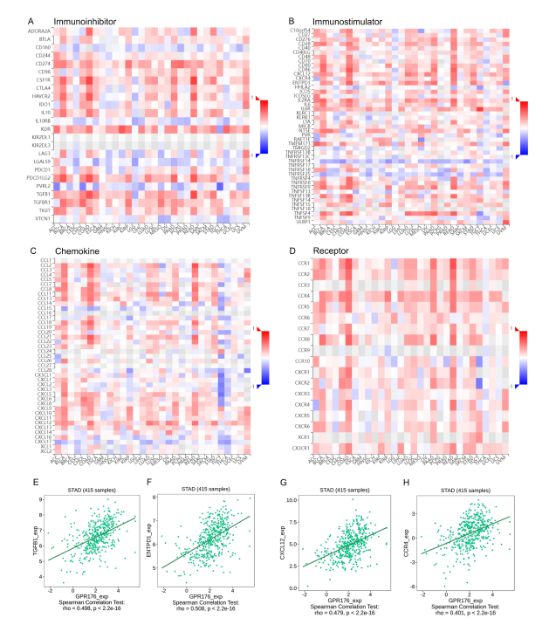


**Supplementary Figure5 GPR176 is related to immunomodulators and chemokines in GC** (A-D) Correlation between GPR176 and immunoinhibitor, immunostimulator, chemokines and chemokines receptors in TISIDB database. (E-H) Relationship between GPR176 and TGFB1, ENTPD1, CXCL12 and CCR4.

Supplementary Figure6


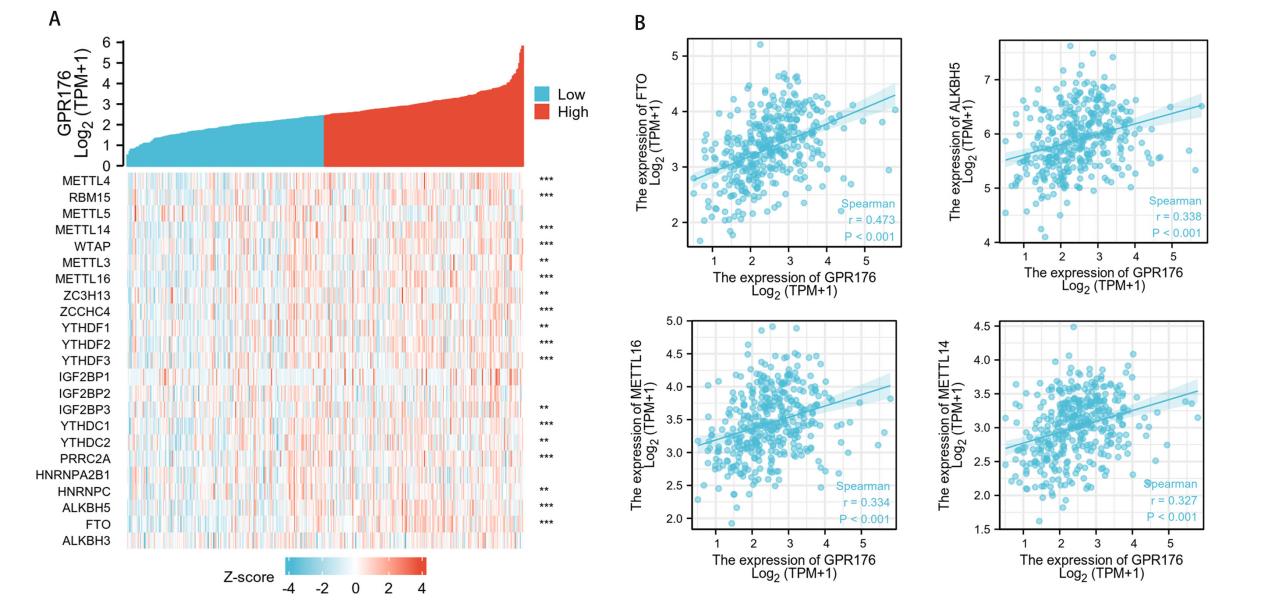


**Supplementary Figure6. Correlation analysis between GPR176 and m6A methylation** (A) Heat map of gene expression related to m6A methylation in different GPR176 expression groups using TCGA data (E-H) Relationship between GPR176 and FTO, ALBKH5, METTL16 and METTL14.

Supplementary Figure7


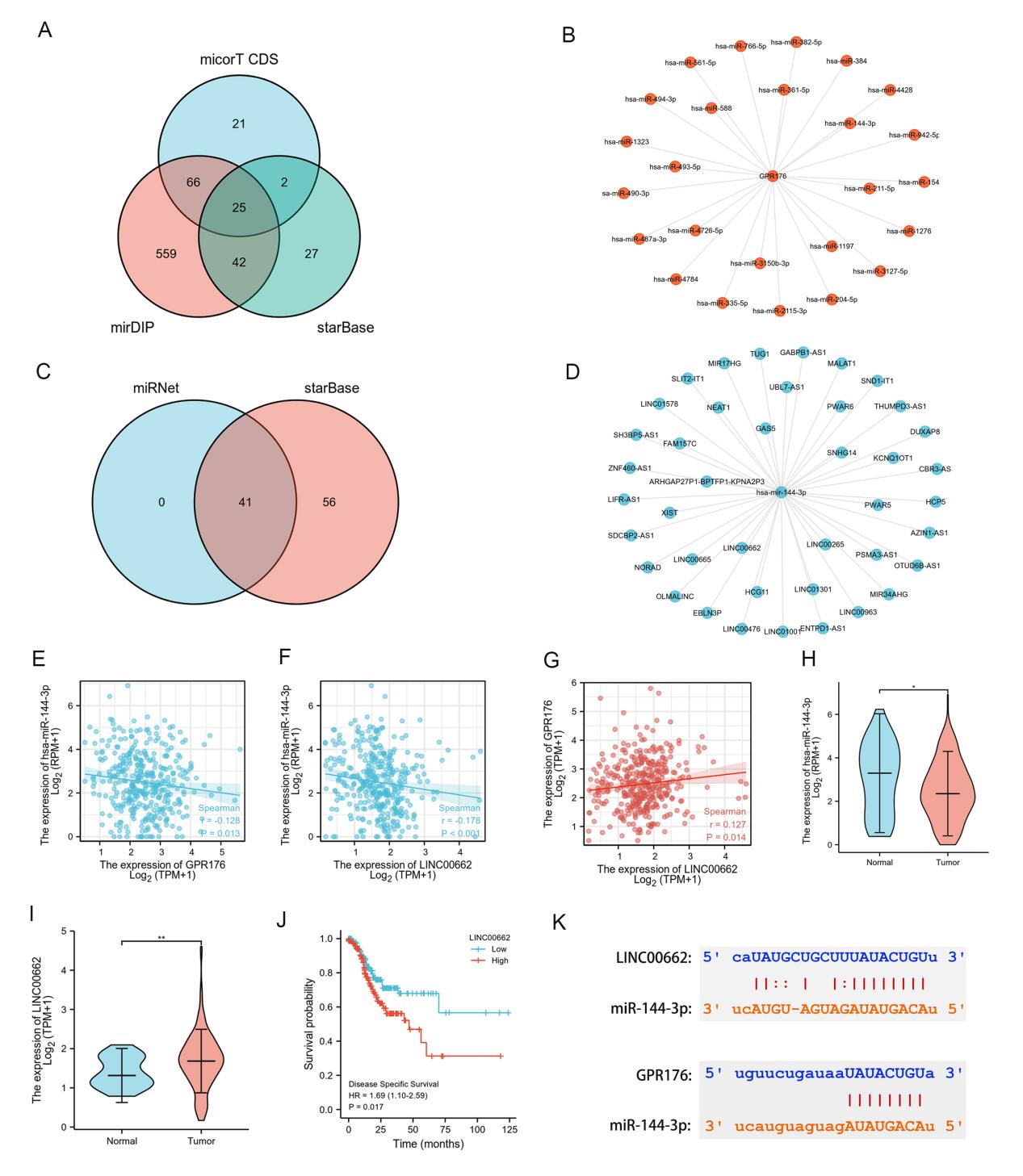


**Supplementary Figure7 Prediction and construction of GPR176 ceRNA network in GC** (A) Venn diagram shows miRNA targeting GPR176 predicted by micorT CDS, mirDIP and starBase. (B) Constructing network diagram based on predicted miRNA (C) Venn diagram shows the lncRNA targeting hsa-miR-144-3p predicted by starBase and miRNet. (D) Constructing ceRNA network (E-G) Scatter plots show results with expressional correlations, including GPR176 and hsa-miR-144-3p, hsa-miR-144-3p and LINC0662, LINC0662 and GPR176. (H-I) The expression of hsa-miR-144-3p and LINC00662 in GC (J) Kaplan–Meier curve of GPR176 in STAD (K) The binding sites of hsa-miR-144-3p on GPR176 and LINC00662 (L) The diagram displays the relationship of the final ceRNA network.

Supplementary Figure8


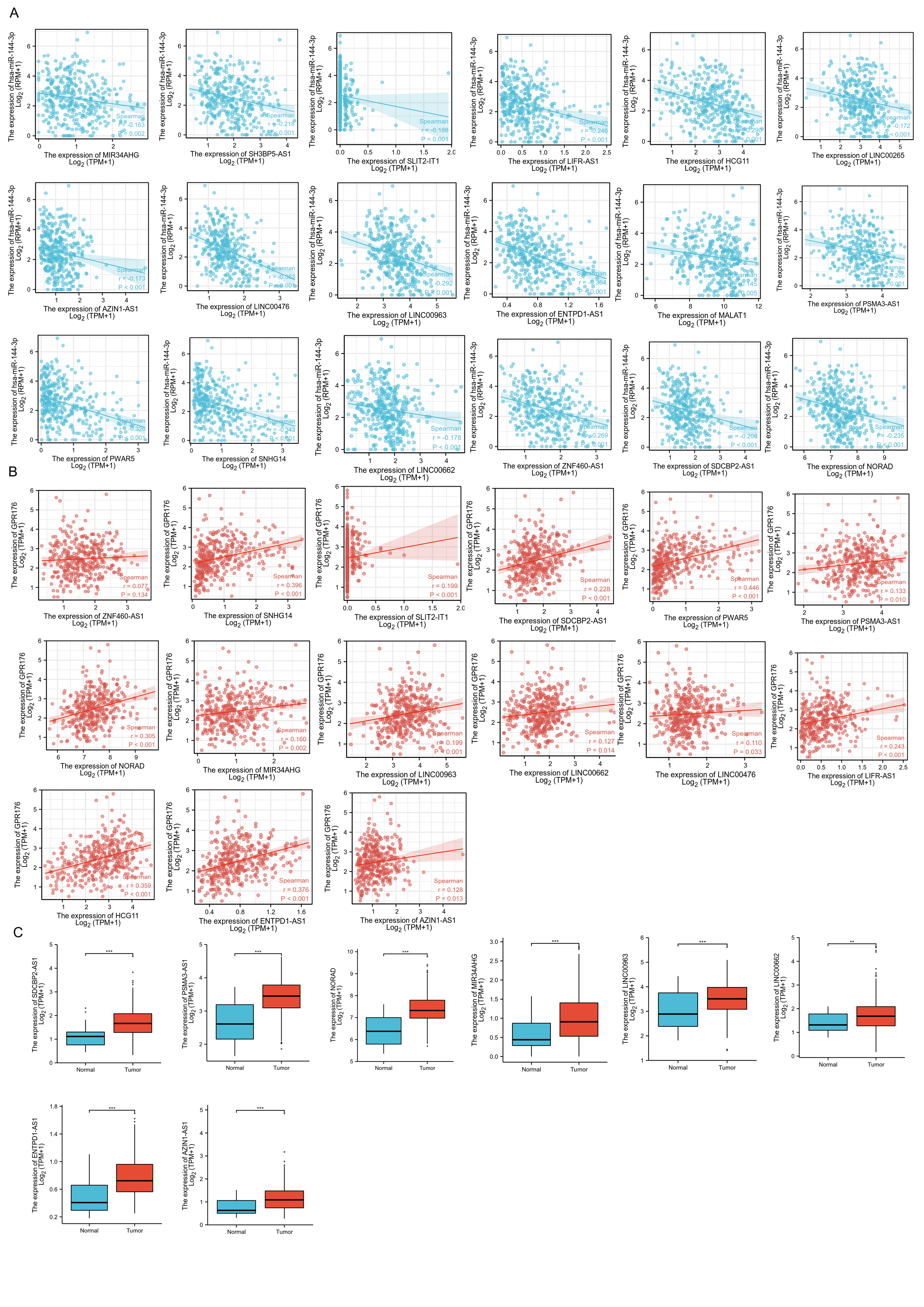


**Supplementary Figure8 Construct LINC00662-has-mir-144-3p-GPR176 ceRNA network in GC.** (A) Correlation of hsa-miR-144-3p with lncRNAs; (B) Correlation of GPR176 with lncRNAs (C) Expression of lncRNAs in gastric cancer
